# Application of first-generation high- and low-dose drug-coated balloons to the femoropopliteal artery disease: a sub-analysis of the POPCORN registry

**DOI:** 10.1186/s42155-023-00390-x

**Published:** 2023-08-10

**Authors:** Masahiko Fujihara, Mitsuyoshi Takahara, Yoshimitsu Soga, Osamu Iida, Daizo Kawasaki, Yusuke Tomoi, Yoshinori Tsubakimoto, Kenji Ogata, Eiji Karashima, Taku Kato, Yohei Kobayashi, Nobuhito Kaneko, Shinya Sasaki, Kei Ichihashi

**Affiliations:** 1 Department of Cardiology, Kishiwada Tokushukai Hospital, 4-27-1, Kamoricho, Kishiwada-City Osaka, 596-8522 Japan; 2 Department of Medicine and Biosystemic Science, Kyushu University Graduate School of Medical Sciences, Fukuoka, Japan; 3 Department of Diabetes Care Medicine, Osaka University Graduate School of Medicine, Suita, Japan; 4 Department of Cardiology, Kokura Memorial Hospital, Kitakyushu, Fukuoka Japan; 5 Cardiovascular Center, Kansai Rosai Hospital, Amagasaki, Japan; 6 Cardiovascular Division, Morinomiya Hospital, Osaka, Japan; 7 Department of Cardiology, Japanese Red Cross Kyoto Daini Hospital, Kyoto, Japan; 8 Department of Cardiology, Miyazaki Medical Association Hospital, Miyazaki, Japan; 9 Department of Cardiology, Shimonoseki City Hospital, Shimonoseki, Yamaguchi, Japan; 10 Department of Cardiology, Rakuwakai Otowa Hospital, Kyoto, Japan; 11 Department of Cardiovascular Center, Osaka Red Cross Hospital, Osaka, Japan; 12 Heart Center, Kasukabe Chuo General Hospital, Saitama, Japan; 13 Department of Cardiology, Saka General Hospital, Miyagi, Japan; 14 Department of Cardiovascular Medicine, Ichinomiya Nishi Hospital, Aichi, Japan

**Keywords:** Femoropopliteal artery, Endovascular Therapy, Drug-Coated Balloon, High-dose DCB, Low-dose DCB

## Abstract

**Background:**

Drug-coated balloons (DCBs) have significantly changed endovascular therapy (EVT) for femoropopliteal artery (FPA) disease, in terms of the expansion of indications for EVT for symptomatic lower extremity arterial disease (LEAD). However, whether there is a difference in the performance among individual DCBs has not yet been fully discussed. The present sub-analysis of real-world data from a prospective trial of first-generation DCBs compared the clinical outcomes between high- and low-dose DCBs using propensity score matching methods. The primary endpoint was the restenosis-free and revascularization-free rates at 1 year.

**Results:**

We compared 592 pairs matched for patient and lesion characteristics using propensity score matching among a total of 2,507 cases with first-generation DCBs (592 and 1,808 cases in the Lutonix low-dose and In.PACT Admiral high-dose DCB groups, respectively). There were no differences in patient/lesion characteristics, procedural success rates, or complications between the two groups. First-generation low-dose DCB had significantly lower patency (73.3% [95% confidence interval, 69.6%–77.3%] in the low-dose DCB group versus 86.2% [84.1%–88.3%] in the high-dose DCB group; *P* < 0.001) and revascularization-free (84.9% [81.9%–88.1%] versus 92.5% [90.8%–94.1%]; *P* < 0.001) rates. Chronic kidney disease on dialysis, cilostazol use, anticoagulant use, and severe calcification had a significant interaction effect in the association (all *P* < 0.05).

**Conclusions:**

EVT to FPA with first-generation DCBs had inferior low-dose patency outcomes as compared with high-dose outcomes in the present cohort.

**Level of evidence:**

Sub analysis of a prospective multicenter study.

## Background

Endovascular therapy (EVT) has been widely applied for symptomatic lower extremity arterial disease (LEAD) of the femoropopliteal artery (FPA) because of its less invasiveness, and it is supported by a large amount of evidence and guidelines [1–3]. With the understanding of the disease, lesion assessment, and technological innovations of the last decade, the EVT results in this area have dramatically improved. Particularly, drug-coated balloon (DCB) therapy has proven effective as a non-stenting treatment and has demonstrated good patency, benefiting many patients [4, 5]. The introduction of DCBs has greatly impacted this field, and a number of DCBs have entered the market. DCBs have several characteristics, including the type of drug and its dose, recipient, and balloon in which the drug is mounted. However, it is still unclear whether all DCBs will equally benefit the therapeutic strategy for FPA. The differences probably have a direct impact on the outcomes. Particularly, the amount of drug and recipient have been reported as important factors.

The POPCORN registry, a recent large-scale study of first-generation DCBs in real-world patient populations, reported that a multiple regression analysis identified several negative determinants of the loss of patency of DCB [6]. The use of low-dose DCB was one of the seven negative determinants. Drug doses in particular have also been an issue with concerns of increased mortality with paclitaxel, and it is still unclear which type or lesions or patient group it will affect [7]. Multiple regression analysis the current study aimed to compare the clinical outcomes between first-generation low- and high-dose DCBS for symptomatic FPA lesions, using the propensity score-matching method.

## Method

### Study population

The current study used a clinical database of the PrOsPective multiCenter registry Of dRug-coated ballooN for femoropopliteal disease (POPCORN) [6]. The POPCORN is an ongoing prospective multicenter observational study that registered adult patients (aged ≥ 20 years) undergoing DCB treatment for femoropopliteal lesions of symptomatic peripheral artery disease (Rutherford categories 2–5) [8] at 81 cardiovascular centers across Japan. Altogether, 2507 patients were registered between March 2018 and December 2019, and 5-year follow-ups have been scheduled. Only the following two first-generation DCBs were used in this study, because no other DCBs are commercially available: low- (Lutonix DCB, Bard, New Hope, MN, USA) and high-dose (IN.PACT Admiral, Medtronic, Santa Rosa, CA, USA) DCBs.

The study was conducted in accordance with the guidelines stipulated in the Declaration of Helsinki and was approved by the institutional review boards of the participating centers. Informed consent was obtained from the participants or, if not possible, from their families. The current study utilized the registry’s 1-year database. In patients with multiple FPA lesions treated, the first registered lesion was selected as their representative.

### Outcome measures

The primary outcome measure was freedom from restenosis, which was compared between the first-generation high- and low-dose DCBs. Restenosis was defined as > 2.4 times of the peak systolic velocity ratio on duplex ultrasound or > 50% of the arterial diameter measured by angiography [9]. The secondary outcome measures included blood flow and severe dissection defined as grade D or severer [10] after DCB treatment, bailout stenting, postoperative ankle–brachial index (ABI), perioperative complication, freedom from reintervention, limb salvage rate, and overall survival.

### Statistical analysis

Data on baseline characteristics are presented as the mean ± standard deviation (SD) and percentage for continuous and discrete variables, respectively, if not otherwise mentioned. A *P* value of < 0.05 was considered statistically significant, and 95% confidence intervals were reported where appropriate. The differences in baseline characteristics between the low-dose and high-dose DCB groups were crudely tested by the Welch’s t and chi-squared tests for continuous and discrete variables, respectively.

When the clinical outcomes were compared between the two groups, propensity score matching was adopted to minimize the intergroup difference in baseline characteristics. The propensity score was developed using the logistic regression model that included the following variables: age, sex, mobility, smoking, diabetes mellitus, renal function, chronic heart failure, medications, Rutherford classification, ankle–brachial index(ABI), aortoiliac revascularization, below-the-knee (BTK) runoff, history of revascularization, popliteal lesion, reference vessel diameter, lesion length, severe calcification (defined as peripheral artery calcification scoring system (PACSS) grade 4) [11], and chronic total occlusion. Matching was performed on the logit of the propensity score within the caliper of 0.2 SD. To maximize the statistical power to detect intergroup prognostic differences, we extracted as many matched samples in the high-dose DCB group to one in the low-dose DCB group as possible. After matching, the intergroup difference was analyzed with stratification by the pairs, and weighted descriptive statistics are reported. The intergroup balance in the baseline characteristics was assessed with the standardized difference. The proportions of perioperative outcomes were compared between the groups using the conditional logistic regression model. Time-to-events were estimated by using the Kaplan–Meier method and were compared between the two groups by the stratified log rank test. The interaction effect of the baseline characteristics on the association of DCB types with restenosis risk was analyzed using the Cox proportional hazards regression model stratified by the matched pairs. All statistical analyses were performed with R version 4.1.1 (R Development Core Team, Vienna, Austria).

## Results

Among 2507 patients undergoing FPA EVT with DCB for symptomatic LEAD, 602 patients were treated with low-dose DCB, and the remaining 1905 patients were treated with high-dose DCB. The patients’ baseline characteristics are summarized in Table 1. The patient characteristics were generally similar between the two groups, with the exceptions of chronic kidney disease (CKD) and Rutherford category. The low-dose DCB group was more likely to have no BTK runoff, knee artery involvement, and severe calcification as compared to the high-dose DCB group. This difference may be due to the compatibility of the high- and low-dose DCBs with the 6- and 5-Fr sizes, respectively.

**Table 1:** Baseline characteristics of the study population before and after propensity score matching

	Overall population (before matching)				Matched population		
	**Low-Dose DCB** Lutonix	**High-Dose DCB** IN.PACT Admiral	Standardized difference (%)	*P* value	**Low-Dose DCB** Lutonix	**High-Dose DCB** IN.PACT Admiral	Standardized difference (%)
	(*n* = 602)	(*n* = 1905)			(*n* = 592, weighted *n* = 592)	(*n* = 1808, weighter *n* = 592)	
Patient characteristics							
Age (years)	75 ± 9	74 ± 9	1.3	0.79	75 ± 9	74 ± 9	2.7
Male sex	389 (64.6%)	1237 (64.9%)	0.7	0.93	383.0 (64.7%)	383.1 (64.7%)	0.0
Non-ambulatory	99 (16.4%)	224 (11.8%)	13.5	0.003	96.0 (16.2%)	93.8 (15.8%)	1.0
Smoking	114 (18.9%)	403 (21.2%)	5.5	0.26	112.0 (18.9%)	115.2 (19.5%)	1.4
Diabetes mellitus	408 (67.8%)	1231 (64.6%)	6.7	0.17	399.0 (67.4%)	396.2 (66.9%)	1.0
Chronic kidney disease (CKD)				< 0.001			
None	158 (26.2%)	602 (31.6%)	11.9		158.0 (26.7%)	158.1 (26.7%)	0.0
CKD without dialysis	222 (36.9%)	795 (41.8%)	10.0		220.0 (37.2%)	230.9 (39.0%)	3.8
CKD on dialysis	222 (36.9%)	506 (26.6%)	22.2		214.0 (36.1%)	203.1 (34.3%)	3.9
(missing data)	0 (0.0%)	2 (0.1%)	4.6	1.00	0.0 (0.0%)	0.0 (0.0%)	0.0
Heart failure	132 (21.9%)	330 (17.3%)	11.6	0.013	126.0 (21.3%)	123.1 (20.8%)	1.2
(missing data)	0 (0.0%)	1 (0.1%)	3.2	1.00	0.0 (0.0%)	0.0 (0.0%)	0.0
Aspirin use	466 (77.5%)	1498 (79.3%)	4.3	0.39	459.0 (77.7%)	458.7 (77.6%)	0.1
(missing data)	1 (0.2%)	16 (0.8%)	9.5	0.14	1.0 (0.2%)	1.0 (0.2%)	0.1
P2Y12 inhibitor use	520 (86.5%)	1634 (86.2%)	1.0	0.89	512.0 (86.6%)	513.3 (86.9%)	0.7
(missing data)	1 (0.2%)	9 (0.5%)	5.4	0.50	1.0 (0.2%)	1.2 (0.2%)	0.8
Cilostazol use	150 (25.0%)	473 (25.3%)	0.7	0.93	149.0 (25.2%)	148.8 (25.2%)	0.1
(missing data)	1 (0.2%)	32 (1.7%)	15.9	0.008	1.0 (0.2%)	1.4 (0.2%)	1.4
Anticoagulant use				0.029			
None	484 (80.4%)	1566 (82.7%)	6.0		479.0 (80.9%)	482.3 (81.5%)	1.4
Warfarin use	61 (10.1%)	130 (6.9%)	11.7		56.0 (9.5%)	52.4 (8.9%)	2.1
DOAC use	57 (9.5%)	197 (10.4%)	3.1		57.0 (9.6%)	57.2 (9.7%)	0.1
(missing data)	0 (0.0%)	12 (0.6%)	11.3	0.11	0.0 (0.0%)	0.0 (0.0%)	0.0
Statin use	354 (59.1%)	1160 (61.6%)	5.1	0.30	347.0 (58.9%)	348.4 (59.1%)	0.5
(missing data)	3 (0.5%)	22 (1.2%)	7.3	0.24	3.0 (0.5%)	3.1 (0.5%)	0.2
Limb characteristics							
Rutherford classification				< 0.001			
Category 2	138 (22.9%)	509 (26.7%)	8.8		137.0 (23.1%)	122.6 (20.7%)	5.9
Category 3	210 (34.9%)	864 (45.4%)	21.5		209.0 (35.3%)	237.4 (40.1%)	9.9
Category 4	63 (10.5%)	188 (9.9%)	2.0		63.0 (10.6%)	67.2 (11.4%)	2.3
Category 5	191 (31.7%)	344 (18.1%)	32.0		183.0 (30.9%)	164.8 (27.8%)	6.7
Ankle–brachial index	0.62 ± 0.23	0.60 ± 0.23	7.5	0.11	0.62 ± 0.23	0.62 ± 0.24	2.9
(missing data)	26 (4.3%)	35 (1.8%)	14.4	0.001	22.0 (3.7%)	20.8 (3.5%)	1.1
Aortoiliac lesion	140 (23.3%)	418 (22.3%)	2.3	0.66	138.0 (23.3%)	138.5 (23.4%)	0.2
(missing data)	0 (0.0%)	30 (1.6%)	17.9	0.004	0.0 (0.0%)	0.0 (0.0%)	0.0
No below-the-knee runoff	103 (17.1%)	218 (11.5%)	16.1	< 0.001	98.0 (16.6%)	100.0 (16.9%)	0.9
(missing data)	0 (0.0%)	6 (0.3%)	7.9	0.37	0.0 (0.0%)	0.0 (0.0%)	0.0
Lesion characteristics							
History of EVT				0.021			
None (de novo)	436 (72.4%)	1455 (76.4%)	9.1		431.0 (72.8%)	434.1 (73.3%)	1.2
1 EVT	87 (14.5%)	274 (14.4%)	0.2		86.0 (14.5%)	84.6 (14.3%)	0.7
≥ 2 EVTs	79 (13.1%)	176 (9.2%)	12.3		75.0 (12.7%)	73.3 (12.4%)	0.9
In-stent restenosis	77 (12.8%)	292 (15.4%)	7.4	0.14	76.0 (12.8%)	78.6 (13.3%)	1.3
(missing data)	0 (0.0%)	3 (0.2%)	5.6	0.77	0.0 (0.0%)	0.0 (0.0%)	0.0
Popliteal lesion	242 (40.2%)	542 (28.5%)	24.9	< 0.001	233.0 (39.4%)	226.3 (38.2%)	2.3
Distal reference vessel diameter (mm)	4.8 ± 0.9	4.9 ± 0.9	1.3	0.78	4.8 ± 0.9	4.8 ± 0.9	0.7
(missing data)	0 (0.0%)	18 (0.9%)	13.8	0.034	0.0 (0.0%)	0.0 (0.0%)	0.0
Lesion length (cm)	13.4 ± 9.1	13.8 ± 9.7	4.2	0.36	13.5 ± 9.2	13.6 ± 9.6	1.3
(missing data)	0 (0.0%)	1 (0.1%)	3.2	1.00	0.0 (0.0%)	0.0 (0.0%)	0.0
Severe calcification (PACSS grade 4)	117 (19.5%)	238 (12.5%)	19.1	< 0.001	111.0 (18.8%)	108.7 (18.4%)	1.0
(missing data)	1 (0.2%)	0 (0.0%)	5.8	0.54	0.0 (0.0%)	0.0 (0.0%)	0.0
Chronic total occlusion (CTO)	152 (25.2%)	531 (27.9%)	6.0	0.22	149.0 (25.2%)	152.6 (25.8%)	1.4
(missing data)	0 (0.0%)	3 (0.2%)	5.6	0.77	0.0 (0.0%)	0.0 (0.0%)	0.0
Intravascular ultrasound use	404 (72.0%)	1328 (73.4%)	3.1	0.55	400.0 (72.1%)	397.3 (73.3%)	2.8
(missing data)	41 (6.8%)	96 (5.0%)	7.5	0.12	37.0 (6.2%)	39.5 (6.7%)	1.7

Data before matching are percentages and means ± standard deviations for discrete and continuous variables, respectively. Data after matching are weighted percentages and weighted means ± weighted standard deviations for discrete and continuous variables, respectively

During a median follow-up period of 14.2 (interquartile range, 10.323.0) months, restenosis was observed in 645 patients. The propensity score matching extracted 592 pairs (592 cases for the low-dose DCB group and 1808 cases in the high-dose DCB group), with no remarkable intergroup difference in baseline characteristics (Table 1). Table 2 shows the perioperative outcomes in the respective groups. There were no differences in post-EVT blood flow, severe dissection defined as grade D, post-procedure　ABI, or procedure-related complications between the two groups. Bailout stent rates were significantly different; however, the rates were very low in both groups; thus, we believe that this difference has a little impact on the main objective of this analysis.

**Table 2:** Perioperative outcomes of the propensity score-matched population

	Low-Dose DCB Lutonix	High-Dose DCB IN.PACT Admiral	*P* value
	(*n* = 592, weighted *n* = 592)	(*n* = 1808, weighter *n* = 592)	
Normal blood flow after DCB	566.0 (95.8%)	565.9 (95.8%)	0.93
(missing data)	1.0 (0.2%)	1.0 (0.2%)	0.92
Dissection grade D or severer	29.0 (4.9%)	24.4 (4.1%)	0.41
(missing data)	0.0 (0.0%)	0.2 (0.0%)	1.00
Bailout Stenting	33.0 (5.6%)	18.1 (3.1%)	0.005
(missing data)	0.0 (0.0%)	0.9 (0.2%)	1.00
Ankle–brachial index after the procedure	0.89 ± 0.18	0.89 ± 0.17	0.36
(missing data)	24.0 (4.1%)	23.3 (3.9%)	0.59
Perioperative complication	18.0 (3.1%)	27.8 (4.8%)	0.20
(missing data)	9.0 (1.5%)	10.1 (1.7%)	0.98
Perioperative death	4.0 (0.7%)	8.8 (1.5%)	0.24
Target lesion revascularization (EVT)	3.0 (0.5%)	5.2 (0.9%)	0.38
Target lesion revascularization (Bypass)	0.0 (0.0%)	0.5 (0.1%)	1.00
Distal embolism	0.0 (0.0%)	5.5 (1.0%)	1.00
Transfusion for bleeding	5.0 (0.9%)	3.0 (0.5%)	0.22
Acute occlusion	3.0 (0.5%)	4.4 (0.8%)	0.58
Vessel rupture	1.0 (0.2%)	0.3 (0.1%)	0.38
Blue toe syndrome	1.0 (0.2%)	0.8 (0.1%)	0.82
Major amputation	0.0 (0.0%)	2.6 (0.4%)	1.00
Myocardial infarction	2.0 (0.3%)	0.8 (0.1%)	0.27
Stroke	1.0 (0.2%)	0.8 (0.1%)	0.95
Renal impairment	1.0 (0.2%)	1.6 (0.3%)	0.82

Data are weighted percentages and weighted means ± weighted standard deviations for discrete and continuous variables, respectively

### Primary and secondary outcome measures

**Fig. 1:**
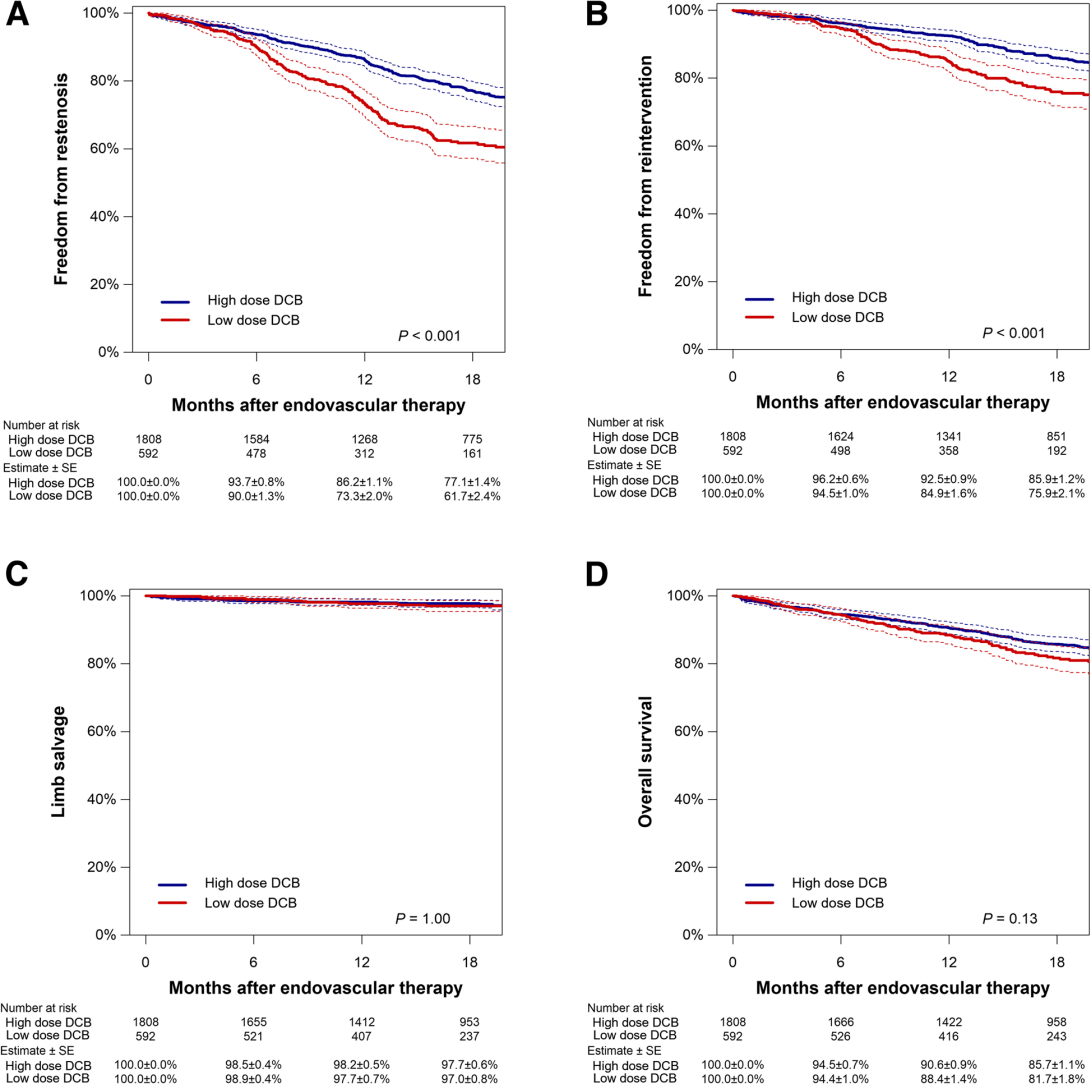
Freedom from restenosis (**A**), freedom from reintervention (**B**), limb salvage (**C**), and overall survival (**D**) in the matched population. Dotted lines indicate 95% confidence intervals. SE, standard error

**Fig. 2:**
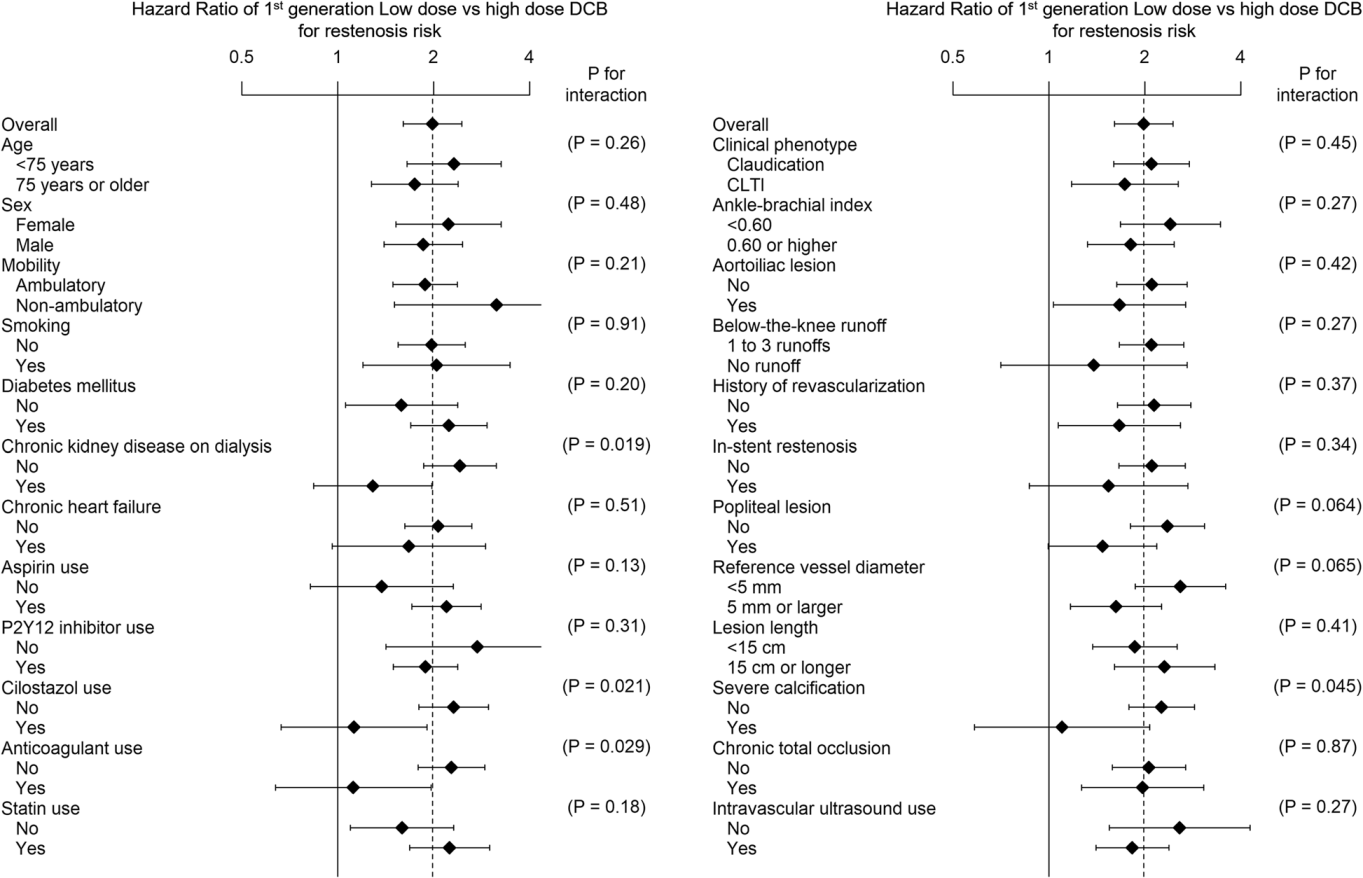
Interaction effect of the baseline characteristics on the association of DCB types with restenosis risk in the matched population. Data are derived from the Cox proportional hazards regression model stratified by the matched pairs. Error bars indicate 95% confidence intervals

As shown in Fig. 1A and B, after the propensity score matching analysis, the low-dose DCB group had significantly lower rates of freedom from restenosis (73.3% [95% confidence interval, 69.6%–77.3%] in the low-dose DCB group versus 86.2% [84.1%–88.3%] in the high-dose DCB group; *P* < 0.001) and reintervention (84.9% [81.9%–88.1%] versus 92.5% [90.8%–94.1%]; *P* < 0.001) at 1 year. Limb salvage and overall survival rates were not significantly different between the two groups (both *P* > 0.05) (Fig. 1C and D). The interaction effect of the baseline characteristics with restenosis risk is shown in Fig. 2. CKD on dialysis, cilostazol use, anticoagulant use, and severe calcification had a significant interaction effect in the association (all *P* < 0.05); the restenosis risk of low-dose DCB versus high-dose DCB was significantly attenuated in the subgroups with those factors, as compared with the that of subgroups without those factors.

## Discussion

This study compared the endovascular approach of FPA lesions for symptomatic LEAD using first-generation high- and low-dose DCBs. Propensity score matching analysis showed that high-dose DCBs performed better within the study period with statistical significance.

There are several explanations for this. The DCB is composed of three components (drug, recipient, and balloon). In addition to its characteristics, the operator selects the DCB that best suits the patient and lesion based on system size (0.014, 0.018, and 0.035 inch) and compatible sheath size (5 or 6 Fr), device diameter, and length.

Both DCBs used in this study were paclitaxel, but their drug-loading doses differed. In.PACT is classified as high-dose DCB (3.5 μg/mm-) and Lutonix as low-dose (2.0 μg/mm^2^). High-dose DCBs may be better for drug residuals in the vessel wall, whereas low-dose DCBs may be better for drug outflow distally. However, the increased mortality risk of paclitaxel devices is known to be independent of the drug dose [12]. Moreover, it the slow flow phenomenon caused by the drug is not associated with worsening chronic limb threatening ischemia (CLTI) [13]. Currently, there is no evidence that high-dose DCBs increase the safety risks, as compared to low-dose DCBs, except for a few small case studies [14, 15].

The results of a recent randomized trial comparing a first-generation high-dose DCB to a second-generation low-dose DCB (Ranger Paclitaxel-Coated PTA Balloon Catheter, Boston Scientific, Marlborough, MA, USA)　did not report any difference in the patency outcomes [16]. Ranger DCBs is classified as low-dose DCBs with a drug dose of 2.0 μg/mm^2^, similar to LUTONIX, but it has an improved recipient TransPax technology, which allows the drug to remain in the vessel wall longer [17]. In other words, the key factor in DCB is the drug amount, but with recipient, even a small amount of drug may be expected to have a greater effect.

Next, the present study confirms that first-generation low-dose DCB is inferior to high-dose DCB on a non-case basis. We evaluated whether the results of the main analysis were independent of case and lesion characteristics using an interaction analysis.

The interaction analysis revealed several interesting points. Although most parameters showed the advantage of the high-dose DCB, the following parameters showed no difference between the high and low-dose DCBs, in the rates of renal failure on dialysis, cilostazol usage, anticoagulant usage, and severe calcification. Renal function and dialysis patients are known to have a high rate of calcification [18], and DCB has been reported to be ineffective for severe calcification [19]. This suggests that both groups had poor outcomes. However, the individual subgroups have smaller sample sizes than the overall population. Therefore, it remains unclear whether there is really no difference or if the small sample size prevents us from obtaining a significant difference. In other words, it is inconclusive whether low-dose DCB is equivalent to high-dose DCB for dialysis, cilostazol, anticoagulants, and PACSS 4 severe calcification. The only thing that can be said about the interaction analysis is that the degree of the inferiority of the low-dose DCB relative to the high-dose DCB is significantly smaller in the subgroup with these four parameters than in the subgroup without these factors. Conversely, in the subgroup without these four factors, the hazard ratio in Fig. 2 is significantly > 1.0. However, there is no guarantee that the low- and high-dose DCB groups are perfectly matched in these subgroups (they are matched only in the total number of cases); thus, we cannot be sure whether this is truly a sign of inferiority of the low-dose DCB group or whether the bias in the low-dose DCB group is associated with an increased risk of restenosis. This is the limiting factor in this study.

## Conclusions

In this study cohort, first-generation low-dose DCB had a significantly lower restenosis-free (73.3% [95% confidence interval, 69.6% to 77.3%] in the low-dose DCB group versus 86.2% [84.1% to 88.3%] in the high-dose DCB group; *P* < 0.001) and revascularization (84.9% [81.9% to 88.1%] versus 92.5% [90.8% to 94.1%]; *P* < 0.001) rates than the first-generation high-dose DCB. Some interaction factors were also observed, but these factors need to be examined in more detail in future studies.

## Data Availability

The datasets used and/or analyzed during the current study are available from the corresponding author on reasonable request.

## Abbreviations

EVT: Endovascular therapy (EVT)
LEAD: Lower extremity arterial disease (LEAD)
FPA: Femoropopliteal artery (FPA)
DCB: Drug-coated balloon (DCB)
BTK: Below-the-knee (BTK)
CLTI: Chronic limb-threatening ischemia (CLTI)
ABI: Ankle-brachial index (ABI)
PACSS: Peripheral artery calcification scoring system (PACSS)
